# Severe Acute Respiratory Syndrome Coronavirus 2-Induced Neurological Complications

**DOI:** 10.3389/fcell.2020.605972

**Published:** 2020-12-10

**Authors:** Shijia Yu, Mingjun Yu

**Affiliations:** ^1^Department of Neurology, Shengjing Hospital of China Medical University, Shenyang, China; ^2^Department of Neurosurgery, Shengjing Hospital of China Medical University, Shenyang, China

**Keywords:** coronavirus, COVID-19, SARS-CoV-2, neurological complications, inflammation, ACE2

## Abstract

Our review aims to highlight the neurological complications of severe acute respiratory syndrome coronavirus 2 (SARS-CoV-2) infection and the available treatments according to the existing literature, discussing the underlying mechanisms. Since the end of 2019, SARS-CoV-2 has induced a worldwide pandemic that has threatened numerous lives. Fever, dry cough, and respiratory symptoms are typical manifestations of COVID-19. Recently, several neurological complications of the central and peripheral nervous systems following SARS-CoV-2 infection have gained clinicians' attention. Encephalopathy, stroke, encephalitis/meningitis, Guillain–Barré syndrome, and multiple sclerosis are considered probable neurological signs of COVID-19. The virus may invade the nervous system directly or induce a massive immune inflammatory response via a “cytokine storm.” Specific antiviral drugs are still under study. To date, immunomodulatory therapies and supportive treatment are the predominant strategies. In order to improve the management of COVID-19 patients, it is crucial to monitor the onset of new neurological complications and to explore drugs/vaccines targeted against SARS-CoV-2 infection.

## Introduction

A global pandemic named coronavirus disease-2019 (COVID-19) broke out at the end of 2019 caused by the novel human coronaviral pathogen, the severe acute respiratory syndrome-coronavirus type 2 (SARS-CoV-2). In the past few months, it has spread rapidly to almost all continents and infected a large number of individuals. According to the updated COVID-19 dashboard from John Hopkins University (Dong et al., 2020), as of November 9, 2020, over 50 million cases were confirmed in 215 countries, with 1,259,245 reported deaths. Data shows that the United States currently has the highest number of confirmed cases and death rate, followed by India and Brazil. In Europe, most cases have been confirmed in France and Spain, while the death rate in Italy is the highest (Dhama et al., 2020a).

The COVID-19 pandemic is challenging healthcare systems around the whole world. SARS-CoV-2 transmits both zoonotically and human-to-human and can spread via respiratory aerosol and direct contact with contaminated surfaces. COVID-19 patients' age could range from 20s to 80s. In particular, older or immune-comprised individuals with preexisting diseases have high risks of viral infection (Chen et al., 2020; Zhang J. J. et al., 2020). Although the general infection fatality risk merely ranges from 0.3 to 0.6%, patients with high viral loads are more prone to dying. Even though scientists and clinicians have spared no effort on SARS-CoV-2 research, there is no proven effective therapy yet. People are recommended to work from home and required to engage in social distancing, quarantine, and isolation to reduce the spread. Businesses have been forced to shut down during periods of 2020, causing severe consequences on global finances.

Human coronaviruses are a large group of enveloped, single-stranded, positive-sense RNA viruses, which can induce acute infections in respiratory and digestive systems. According to the full sequence published in January 2020, SARS-CoV-2 belongs to a beta genus of coronaviruses, sharing ~80% similarity with SARS-CoV (Andersen et al., 2020; Wu et al., 2020). In addition, SARS-CoV-2 possesses over 90% homology with coronaviruses from the bat and the pangolin, which explains its potent cross-species transmitting ability (Koralnik and Tyler, 2020). The virus is composed of four classic proteins: spike (S) glycoprotein, envelope (E) glycoprotein, membrane (M) glycoprotein, and nucleocapsid (N) protein (Lin et al., 2003). Viral detection depends on the conserved N protein, while the S glycoprotein functions as a trimeric fusion to determine the tropism of virus (Almazan et al., 2000). Intriguingly, specific domains encoding polybasic cleavage sites are detected in the S protein of SARS-CoV-2, which may lead its stronger infectiousness (Andersen et al., 2020).

Generally, patients with COVID-19 develop flu-like symptoms such as fever, fatigue, and dry cough (Bhatraju et al., 2020; Guan et al., 2020; Huang et al., 2020). Gastroenteritis, diarrhea, and myocarditis have also been reported in previous studies (Huang et al., 2020). Moreover, around 10% are asymptomatic or present mild pneumonia only (Guan et al., 2020). Some patients with confirmed SARS-CoV-2 infection only complain of weakness, dizziness, or neurological disorders without any respiratory symptoms (Favas et al., 2020). Analyses of the COVID-19-associated neurological manifestations and explorations of the underlying mechanisms are needed for a better understanding of the disease. Here, we present a review of the previously reported cases of various neurological complications associated with SARS-CoV-2 infection and discuss the possible pathogenesis.

## Pathology of COVID-19

According to former studies, SARS-CoV-2 can invade multiple organs and rapidly replicate to activate inflammatory responses in the human body (Dhama et al., 2020b). Critical illness may progress to acute respiratory distress syndrome (ARDS) or acute respiratory failure. A study of a series of 135 cases reported a high prevalence of lymphopenia (75.4%) and eosinopenia (52.9%) concurrent with SARS-CoV-2 infection (Zhang J. J. et al., 2020). Leukopenia happens more frequently in adult patients, whereas children are prone to leukocytosis and upregulated creatine kinase (Han et al., 2020). D-dimer, procalcitonin, and C-reactive protein are notably increased in patients with high viral loads (Zhang J. J. et al., 2020). Patients may have decreased platelet count and increased blood urea nitrogen (Mao et al., 2020). Severe cases may develop liver damage with elevated transaminases (Dhama et al., 2020b). As reported, CT scans may demonstrate ground-glass opacities, traction bronchiectasis, or even bilateral multilobar consolidation in the lungs (Zhu et al., 2020). Neurological manifestations of SARS-CoV-2 infection can be induced by direct invasion of the nervous system or by inflammatory responses (Alshebri et al., 2020). Affecting either the neurons or the glia cells can lead to neurological pathologies (Ibrahim, 2020).

## Neurological Complications of COVID-19

Neurological manifestations could occur in the central nervous system (CNS), peripheral nervous system (PNS), and relative skeletal muscles (Kutlubaev, 2020; Mao et al., 2020; Puccioni-Sohler et al., 2020). A systematic review about 290 patients revealed that 91% of patients with neurological discomfort present with CNS symptoms, while only PNS symptoms account for 9% (Ibrahim, 2020). Headaches and dizziness are regarded as the most common symptoms in CNS, while smell and taste disorders in PNS (Padda et al., 2020). A retrospective study about 213 patients from Italy with positive SARS-CoV-2 testing results indicates that headache (4.6%), fatigue (32.3%), myalgia (9.3%), anosmia (6.1%), seizure (2.8%), and consciousness disorder (40.3%) are common symptoms accompanied with fever or dyspnea in COVID-19 (Luigetti et al., 2020). Eighteen out of thirty six critically ill cases had consciousness disordered in Japan (Kohara and Kawamoto, 2020).

### Neurological Symptoms Induced by Systemic Inflammation

Encephalopathy is considered the most common CNS complication of COVID-19 (caused by hypoxia or systemic diseases) (Kholin et al., 2020). About 50% of the hospitalized COVID-19 cases develop encephalopathy (Nath and Smith, 2020). If the patients' temperature surpasses 39.5°C or if blood oxygen saturation falls below 85%, it can lead to dizziness, ataxia, mental disorder, cognitive dysfunction, or even consciousness impairment. Older or immunosuppressed patients with cardiovascular, hepatic, and renal comorbidities are more susceptible to encephalopathy (Mao et al., 2020). Generally, once the primary diseases are addressed, the neurological impairments can be partially or completely reversible. Based on a survey of 304 COVID-19-confirmed patients including 108 critical patients, there is no evidence of additional risks for acute symptomatic seizure or status epilepticus (Lu et al., 2020). Nevertheless, some individuals manifest sudden seizure due to high fever. For patients with previously diagnosed epilepsy, sleep disorders and apprehension related to the unprecedented worldwide COVID-19 pandemic may pose extra trigger for onset of seizure (Parihar et al., 2020). Four SARS-CoV-2-positive cases in their 60s−70s presented with seizures, and two of them had electroencephalogram (EEG)-indicated epilepsy (Parauda et al., 2020). They were diagnosed with posterior reversible encephalopathy syndrome (PRES), with cerebral angioedema. In addition, a 74-year-old COVID-19 patient manifested altered mental status with epileptiform discharges in his temporal lobe (Filatov et al., 2020). Postmortem evaluation was conducted in four COVID-19-confirmed patients to reveal the hypoxia-induced cerebral impairments. There were multiple microhemorrhages and enlarged space around the vessels. Leukocytes infiltrated the myelin sheaths and small perivascular lesions appeared in the white matter (Kantonen et al., 2020). Moreover, researchers have recognized abnormal fluid-attenuated inversion recovery (FLAIR) signals in the neuroimaging scans of 44% (12/27) ICU-admitted patients who presented with neurological complications (Kandemirli et al., 2020). Kremer et al. illustrated the neuroradiological patterns in the brain MRIs of 37 COVID-19 patients, revealing multifocal FLAIR/T2 hyperintensities with contrast enhancement, and extensive microhemorrhagic abnormalities in the cerebral white matter (Kremer et al., 2020). Pediatric multisystem inflammatory syndrome (PIMS) induced by SARS-CoV-2 could affect the nervous system as well (Padda et al., 2020). Children may present with headache, weakness, hyporeflexia, and brain parenchyma impaired symptoms (Padda et al., 2020). Abnormal imaging signals are evident in the splenium of the corpus callosum (Abdel-Mannan et al., 2020).

Cerebrovascular disease is considered to be another main neurological complication of SARS-CoV-2 infection, which has a high mortality rate (Chua et al., 2020). The pooled prevalence of COVID-19-related acute cerebrovascular disease is inferred as 2.3% (Favas et al., 2020). A Spanish medical center reported that 1.4% of the total 1,683 hospitalized patients with COVID-19 developed cerebrovascular disease (Hernandez-Fernandez et al., 2020). Among them, 73.9% of cases were diagnosed with ischemic stroke, while 21.7% were diagnosed with hemorrhagic stroke with elevated ferritin levels. The vertebrobasilar artery was the most frequently blocked vessel in the ischemic patients. Their brain biopsies evidenced endothelium injury and thrombotic microangiopathy, rather than necrotizing encephalitis or vasculitis. This suggested that severely infected patients are more likely to develop hemorrhagic or ischemic stroke due to coagulopathy (Tang et al., 2020). Patients with cardiocerebrovascular risk factors have a predisposition for acute stroke during COVID-19 dissemination (Li et al., 2020). Six COVID-19 patients with acute ischemic stroke in large vessels developed aphasia, dysarthria, prosopoplegia, paralysis, sensory loss, and even acute confusion. Two cases had multiple infarctions caused by simultaneous venous and arterial thrombosis. Lupus anticoagulants were positive in five patients, suggesting a coagulation disturbance. The angiograms demonstrated occlusion of the posterior or middle cerebral artery trunk or branches (Beyrouti et al., 2020). Oxley et al. admitted five COVID-19 cases with typical neurological dysfunction signs, such as hemiplegia, sensory deficit, facial weakness, or dysarthria, due to infarction in their large vessel territories. They accepted anticoagulant treatments, and one of them improved greatly within 2 weeks (Oxley et al., 2020). In another series of six cases aged from 57 to 82 years old, four developed ischemic stroke and the other two developed hemorrhagic stroke. All of them presented severe pneumonia and complications in multiple organs with elevated transaminases and LDH levels in blood tests, which indicated poor outcome. However, only one patient had possible vessel-related risk factors for stroke before COVID-19 (Morassi et al., 2020). Four SARS-CoV-2-positive patients from New York showed focal diffusion weighted imaging (DWI) hyperintensities in the splenium of the corpus callosum, including two patients with rare isolated lesions. Their mental status was apparently impaired. Three of them progressed to severe inflammatory hypercoagulable status during admission, requiring mechanical ventilation and dialysis. Subarachnoid hemorrhage (SAH) and hemorrhagic transformation after ischemic stroke occurred in two cases. However, SARS-CoV-2 was negative in their CSF (Sparr and Bieri, 2020). Another five cases infected by SARS-CoV-2 developed intracerebral hemorrhage (ICH) during admission. Four of them had ICHs in the lobe area with no cerebral vascular malformation in computed tomography angiography (CTA) examination (Benger et al., 2020). Hemorrhagic lesions may be caused by the coagulopathy derived from severe systemic infection or by the viral invasion of the vascular endothelium (Al Saiegh et al., 2020). In turn, lesions to the cerebral white matter and the blood vessels may aggravate patients' general condition (Tang et al., 2020). Patients with mechanical ventilation support may develop secondary neurological disorders because of the brain-lung crosstalk. The stress state related to hypoxia and to endothelial cell injury can aggravate inflammation, cause hypercoagulability, and lead to “pulmonary encephalopathy”–like impairments (Battaglini et al., 2020).

### Direct Invasion Into the Nervous System

Coronaviruses can directly invade the CNS for their neurotropism, causing encephalitis or meningitis (Alshebri et al., 2020; Serrano-Serrano et al., 2020). Based on the autopsy analyses of 17 COVID-19 patients, 8 individuals were identified SARS-CoV-2 positive in brain tissue with cerebral edema and vascular congestion (Remmelink et al., 2020). A Japanese male developed new-onset seizure and unconsciousness following fever and general weakness. RT-PCR test of SARS-CoV-2 in CSF was positive. Taking these results together with brain MRI results, the patient was likely to suffer from SARS-CoV-2-associated meningitis (Moriguchi et al., 2020). McAbee et al. described a case with encephalitis who was only 11 years old (McAbee et al., 2020). The patient had a sudden status epilepticus and high fever without any preexisting diseases. CSF assay detected positive SARS-CoV-2, together with elevated red and white cells, but normal protein and glucose concentrations. The EEG recorded occasional delta waves in the frontal lobe. A 20-year-old female with COVID-19 presented with mental disorder and urinary incontinence 4 days after flu-like symptoms (Babar et al., 2020). Laboratory assay found predominantly elevated ferritin and D-dimer. No remarkable findings were acquired from MRI and CSF analysis. She received methylprednisolone therapy over 20 days and gained intermittent awareness. Despite absence of direct evidence supporting viral infection in the CSF, the patient was still highly speculated as SARS-CoV-2 encephalitis because the neurological manifestations had significantly improved after SARS-CoV-19 turning negative. Another COVID-19-related encephalitic patient presented with mental disturbances and psychotic features without early respiratory symptoms. MRI detected multiple lesions in the thalamus, cortex, and corpus callosum with abnormal T2/FLAIR signals (Rebeiz et al., 2020). Moreover, brainstem encephalitis was suspected in a 65-year-old woman with SARS-CoV-2 infection (Khoo et al., 2020). She developed involuntary limb movement with myoclonus, nonsense words, diplopia, and disturbed cognition 2 weeks after presenting dry cough and fever. Neurological examination confirmed hypermyotonia on both sides. However, MRI and CSF analyses reported unrepresented results. An experimental treatment with corticosteroids resulted in neurological improvement, which further verified the original speculated diagnosis of postinfectious brainstem encephalitis. The autopsy of an infant who died from COVID-19 revealed a limited distribution of SARS-CoV-2 in choroid plexus, lateral ventricle, and a few regions in the frontal cortex. It is known that viruses and immune factors enter the developing human brain through a weakened blood–brain barrier and induce neural impairments (da et al., 2020). Although not all cases test positive for SARS-CoV-2 in the CSF, signs of nervous system infection or impairment should arouse clinicians' attention to probable neurological complications of COVID-19 (Puccioni-Sohler et al., 2020).

Moreover, chemosensory disorders, including anosmia and ageusia, affect COVID-19 patients (Koralnik and Tyler, 2020; Padda et al., 2020; Vaira et al., 2020). Over one-third of the patients with COVID-19 present with smell or taste disturbances (Favas et al., 2020). These symptoms can develop before, simultaneously, or after cough and fever (Spinato et al., 2020). About 50% of patients report hyposmia or hypogeusia at the initial stage of COVID-19 (Beltran-Corbellini et al., 2020; Cherry et al., 2020). Statistics suggest that dysosmia and dysgeusia are predictors for COVID-19. When considered together with fever, the sensitivity is 70% and the discrimination accuracy is 75% (Roland et al., 2020). The prevalence of smell and taste dysfunctions decreases in older patients (Agyeman et al., 2020), and female patients are more prone to smell disturbance (Meng et al., 2020). Furthermore, about 12% of the COVID-19 patients develop only anosmia as initial symptom (Puccioni-Sohler et al., 2020). Eight cases developed sudden and complete loss of smell without respiratory symptoms (Gilani et al., 2020). Additionally, patients may present with other sensory dysfunctions, including oropharyngeal and ontological/vestibular disturbances: sore throat, dysphagia, vertigo, tinnitus, and hearing loss are common complications (Krajewska et al., 2020; Ozcelik Korkmaz et al., 2020). For instance, a middle-aged woman with sudden dizziness and dry throat was confirmed to have COVID-19. Although her brain MRI showed no abnormal signs, CT demonstrated ground-glass-like infiltration in the lungs (Kong et al., 2020). Careful assessment of otolaryngologic manifestations, combined with nucleic acid and imaging examinations, may provide great help for the early identification of COVID-19.

### Autoimmune-Related Disorders in the Nervous System

SARS-CoV-2 infection can also lead to demyelinating diseases through neurological immune response, both in the CNS and PNS. Guillain–Barré syndrome (GBS) is a common PNS complication caused by an overactivated autoimmune reaction (Wakerley and Yuki, 2013). Progressive flaccid paralysis, dysergia, and areflexia are considered its typical neurological manifestations. The first COVID-19-related GBS patient was reported in January in Wuhan (Zhao et al., 2020). This 61-year-old woman complained of sudden weakness of the lower limbs without history of fever or cough. Lymphocytopenia and thrombocytopenia were determined by laboratory tests. SARS-CoV-2 was positive. CSF results showed an elevation of protein levels but normal cell counts. Electrophysiological examinations detected motor and sensory nerves demyelination with prolonged distal latency and absent F wave, indicating the GBS. Another published series of cases studied five patients with COVID-19-related GBS in Italy (Toscano et al., 2020). These patients presented with facial and/or limb paresis and paresthesia within 5–10 days after the onset of COVID-19 symptoms. CSF tests showed high protein levels without SARS-CoV-2. Antiganglioside antibodies were all negative in the three tested cases. Caudal roots or facial nerve enhancement was observed in MRI, implying an ongoing immune inflammatory response in nerves. Intravenous immunoglobulin (IVIg) was administered to all the patients, but only two improved. Additionally, two Spanish men presented with Miller Fisher syndrome and polycranial neuritis respectively, several days after COVID-19 (Gutierrez-Ortiz et al., 2020). They had diplopia, balance disturbances, and areflexia with albuminocytologic dissociation in the CSF examination. One 50-year-old man presented with right oculomotor palsy and right internuclear ophthalmoparesis. His GD1b-IgG antibodies were positive. Another 39-year-old man developed bilateral palsy of abducens. Both cases were SARS-CoV-2 positive in the oropharyngeal swab sample but negative in CSF. In addition, Dinkin et al. described two COVID-19 cases who developed ophthalmoplegia several days after flu-like symptoms (Dinkin et al., 2020). MRI exhibited enhancement of injured nerves, including the optic nerve and oculomotor nerve. Neither ganglioside antibodies nor SARS-CoV-2 were detected in CSF. Another 51-year-old man presented with limb weakness, sensory disturbances in hands and feet, and areflexia after 2 weeks of fever and cough (Pfefferkorn et al., 2020). Then his condition rapidly deteriorated into ventilator dependence with an almost locked-in syndrome in the PNS. SARS-CoV-2 was positive in a pharyngeal swab test but negative in CSF. Albuminocytologic dissociation was identified in the CSF 13 days later. Electrophysiology revealed demyelination of multiple peripheral nerves, and MRI detected equal enhancement of anterior and posterior nerve roots from different spinal levels. Based on these aforementioned cases, GBS should be regarded as a possible neurological sign of COVID-19, especially in patients with lymphocytopenia. Moreover, GBS with generalized paralysis can further aggravate patients' general conditions.

Multiple sclerosis (MS) is a subacute immune-mediated and demyelinating disease of the CNS. The patients' neurological manifestations often exacerbate and relapse due to infection or stress (Sadeghmousavi and Rezaei, 2020). The mainstay therapy is immunosuppressants. In such cases, patients may suffer from severe lymphopenia with poor prognosis if they are involved in COVID-19 simultaneously (Dersch et al., 2020; Sadeghmousavi and Rezaei, 2020). Additionally, they may develop emerging MS due to post-inflammatory immune response. The first published case recognized as MS with progressively impaired vision due to optic neuritis was reported in May in Spain (Palao et al., 2020). This 29-year-old woman with COVID-19 complained of retro-ocular pain, transient weakness, and myalgia of her limbs with hyperreflexia on admission. MRI demonstrated enhancement of the right optic nerve and demyelinating lesions surrounding the lateral ventricles, while the spinal cord was normal. Lumbar puncture detected oligoclonal bands of IgG but no aquaporin-4 (AQP4) or MOG antibodies. No SARS-CoV-2 was identified in CSF. High-dose steroids were administered and her vision recovered gradually.

Another reported COVID-19-associated neurological complication induced by autoimmunity is acute disseminated encephalomyelitis (ADEM). Patients may present as encephalopathy and transverse myelitis with weakness and numbness of the limbs and urinary disorders. A 51-year-old German woman with COVID-19 lost consciousness and manifested dull oculocephalic reflex (Parsons et al., 2020). Diffused FLAIR hyperintensive signals were detected in the deep brain tissue and white matter adjacent to cortices. The CSF examination showed xanthochromia, with elevated glucose and protein levels. Pathogen assays were negative, but oligoclonal bands were positive in both serum and CSF. Antibodies for AQP4 were absent. All these findings highly supported ADEM in this case. The patient regained consciousness after weeks of IVIg and steroids treatment. Utukuri et al. reported a 44-year-old man with COVID-19 who developed ADEM (Utukuri et al., 2020). He was initially admitted for urinary retention, inability to walk on his own, and back pain, without any symptoms of respiratory infection. MRI showed slightly increased T2 signals in the spinal cord and hyperintensive FLAIR signals in periventricular or juxtacortical regions, indicating ongoing acute lesions associated with demyelination. The CSF analysis revealed upregulated protein levels with normal IgG and absent oligoclonal bands. No SARS-CoV-2 was found in CSF. Additionally, ADEM occurred in a 64-year-old woman with COVID-19 from Italy (Novi et al., 2020). She complained of alternations in the bilateral visual fields, impaired smell and taste, and numbness of the right lower limb. The Babinski sign was induced on the left side. MRI found dissembled abnormalities in both cerebral hemisphere and a segmental injury at the level of the 8th thoracic vertebra of the spinal cord, with typical T1 enhancement. SAR2-CoV-2 was positive in her CSF. Moreover, CSF and serum analyses found oligoclonal bands, but no AQP4 antibody. She received high-dose steroids together with IVIg for 14 days, partially recovering her vision and reducing T1-enhanced lesions. On the contrary, a 71-year-old man, who was positive for COVID-19 after a coronary artery bypass surgery, rapidly deteriorated to multiple organ dysfunction syndrome (MODS) and did not survive (Reichard et al., 2020). The autopsy revealed disseminated focal hemorrhage and edema in the cerebral white matter. Immunohistochemical analysis indicated ADEM-like lesions around vessels. Acute hemorrhagic necrotizing encephalopathy was diagnosed in a woman about 60 years old with positive SARS-CoV-2 (Poyiadji et al., 2020). She manifested altered mental status after several days of fever and cough. No virus was detected in CSF. Brain MRI revealed patchy and bilateral hemorrhagic signals with enhanced edge in the basal ganglia region and medial temporal lobes. IVIg was applied to get generally recovered consciousness. A 59-year-old woman with aplastic anemia developed epilepsy and consciousness disorder 10 days after flu-like symptoms (Dixon et al., 2020). She was highly suspected of having acute necrotizing encephalopathy as a post-immune consequence. SARS-CoV-2 was positive on the nasopharyngeal swab sample. Radiological examinations revealed a fast-progressing condition from brainstem swelling to disseminated hemorrhage. No microorganism was detected in CSF. She did not improve with steroid treatment and died 8 days after admission. Another woman without preexisting diseases was suspected of acute necrotizing myelitis with neck pain and motor and sensory weakness (Sotoca and Rodriguez-Alvarez, 2020). Spinal MRI showed T2 abnormal hyperintensity in a long segment. There was clinical improvement with plasma exchange and methylprednisolone therapies. In addition, a 32-year-old male with COVID-19 developed acute transverse myelitis with paraplegia and dysuria after a 2-day fever (AlKetbi et al., 2020). MRI showed lesions at the cervical and lumbar levels of the spinal cord. Methylprednisolone was administered to relieve his symptoms.

### Other Neurological Complications

Two COVID-19-positive women from New York presented with ophthalmoparesis, headache, and sensory disorder. They both complained of typical diplopia because of abducens nerve palsy. There was neither SARS-CoV-2 RNA nor ganglioside antibodies detected in CSF. The MRI revealed hypothalamic and tegmental mesencephalic hyperintensity in FLAIR and T2 sequences, which is indicative of Wernicke encephalopathy. However, no laboratory evidence supported abnormal thiamine levels (Pascual-Goni et al., 2020).

Additionally, SARS-CoV-2 infection is thought to progress from olfactory bulbs into the diencephalon or the brainstem, causing extrapyramidal symptoms (Mendez-Guerrero et al., 2020; Rabano-Suarez et al., 2020). A 58-year-old man, who previously had good health, was positive for COVID-19 with symptoms of fever, cough, and dyspnea (Mendez-Guerrero et al., 2020). He then developed olfactory disturbances, opsoclonus, bilateral resting myoclonus, consciousness impairment, and asymmetric hypokinetic-rigid syndrome without any metabolic disturbance. Protein level was elevated slightly in CSF. [^123^I]-ioflupane dopamine transporter single-photon emission CT (DaT-SPECT) images indicated that the presynaptic uptake of dopamine in the bilateral putamina reduced asymmetrically. Three patients were diagnosed with generalized myoclonus (Rabano-Suarez et al., 2020). They all felt decreased taste and developed myoclonus in the face, nasopharynx, and arms, with absence of microorganism in CSF. After methylprednisolone and plasma exchange treatment, their general condition improved gradually.

Musculoskeletal manifestations are another common neurological complication, including fatigue or generalized weakness, myalgia or myositis, and skeletal muscle injury. Serum creatine kinase (CK) was significantly increased in 124 out of 213 (58.2%) COVID-19 patients (Luigetti et al., 2020). Myalgias may be associated with the severity of infection (Zhang X. et al., 2020). Researchers investigated nine cases that presented with back pain, dyskinesia, and paresthesia in lower limbs. Spinal cord MRI revealed intramuscular edema in seven patients, suggesting paraspinal myositis (Mehan et al., 2020). Besides, SARS infection could cause functional deficits and myalgias in skeletal muscle with elevated CK level (Xu P. et al., 2020). Patients with SARS infection lost about 32% of their grip strength and shortened about 13% of 6 min walking distance (Lau et al., 2005). Virus-induced inflammatory immune response and deconditioning during severe illness may exacerbate the muscular impairments (Disser et al., 2020). However, more clinical cases are needed to explore whether SARS-CoV-2 can affect musculoskeletal sequelae in long term.

**Figure 1:**
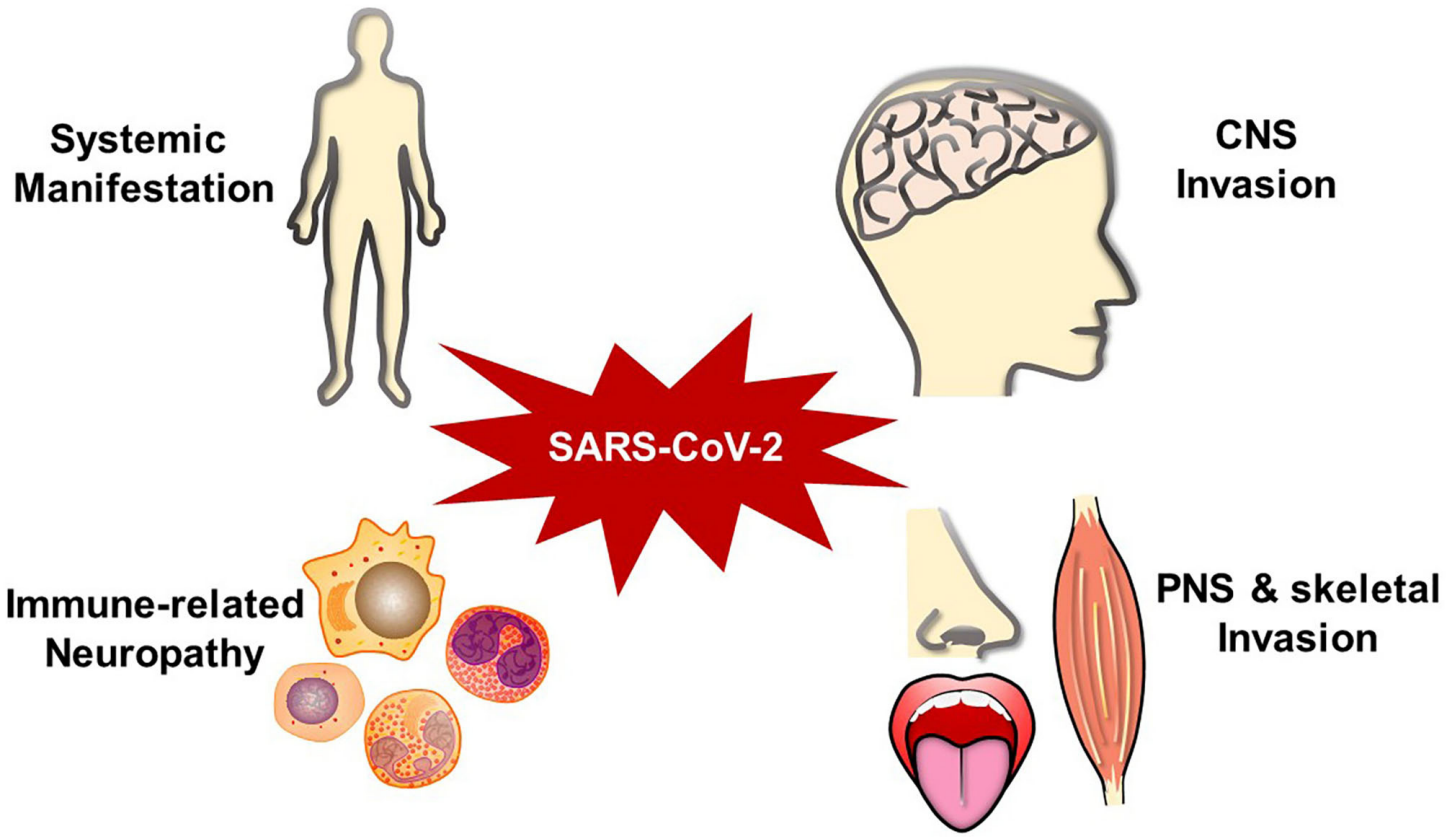
SARS-CoV-2-associated neurological impairments.

As a widely spread pandemic, COVID-19 has influenced thousands of people's lives. In addition to the common respiratory symptoms, patients can develop diverse neurological complications (Figure 1). Patients' neurological manifestations may present as the earliest infected signs (Chua et al., 2020). Individuals may suffer months of malaise, generalized fatigue, or decreased exercise tolerance even after recovered (Nath and Smith, 2020). It is important to differentiate SARS-CoV-2-associated neurological complications from other neurological diseases, such as primary epilepsy, mitochondrial encephalomyopathy, paraneoplastic syndromes, chemosensory disturbances in early neurodegenerative diseases, toxin/drug-related neural injury, and other intracranial infections. Although doctors already pay attention to SARS-CoV-2 impacts in multiple organs, a more comprehensive and deeper understanding should be attained for the complex neurological manifestations. It is crucial to inquire patients about their medical histories, perform the neurological physical examination carefully, and combine with imaging and pathogenic examination results.

## Mechanism of SARS-CoV-2 Infection

**Figure 2:**
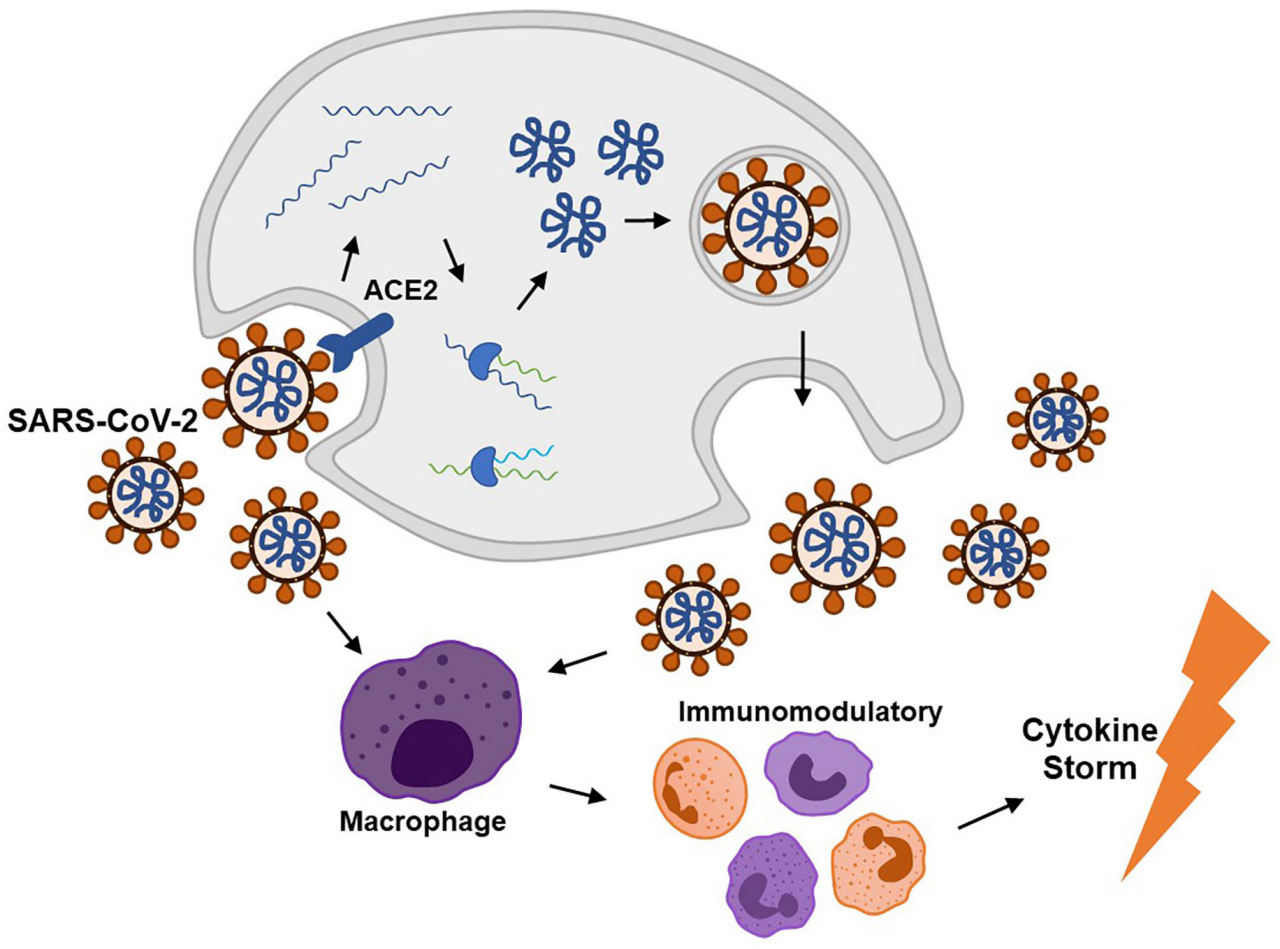
SARS-CoV-2 invades into human cells via the ACE2 receptor and then replicates to cause immunomodulatory disorder through a cytokine storm.

According to previous literatures, SARS-CoV-2 infection can cause complications via direct invasion and immune disturbance through hematogenous or transsynaptic propagation (Figure 2) (Haddadi and Asadian, 2020; Puccioni-Sohler et al., 2020). SARS-CoV-2 is regarded as neurotropic virus. SARS-CoV-2 replicates via synthesizing viral polyproteins and assembling RNA in cells (Shang et al., 2020b). As known, there is an S-protein domain on its surface, with high affinity to angiotensin-converting enzyme 2 (ACE2). ACE2 is widely distributed on the cell membrane in cerebral cortex, vascular endothelium, oral and nasal mucosa (Uhlen et al., 2015; Xu H. et al., 2020). ACE2 receptors are expressed in neurons and glia cells, which may explain the potential neural invasion of SARS-CoV-2 (Baig et al., 2020). Evidence supported that transmembrane protease serine 2 (TMPRSS2) could promote SARS-CoV-2 migration and infection by preactivating S-protein and cleaving ACE2. Endothelial damage and neuronal apoptosis can be induced by SARS-CoV-2 infection (Pranata et al., 2020). ACE2 receptors on vascular endothelial cell membrane may participate in regulating blood pressure, which is closely related to stroke (Tsatsakis et al., 2020; Viana et al., 2020). Also, SARS-CoV-2 may cause vascular endothelial damages, facilitating viral invasion into CNS (Baig et al., 2020). Considering distribution of ACE2 in the muscle system, SARS-CoV-2 could upregulate lactate levels, reduce oxygen/energy supply, and cause myalgia (Kucuk et al., 2020). Besides, virus could spread directly into the brain through the cribriform plate to invade cranial nerves. Bryche et al. studied the SARS-CoV-2-induced deficits of the olfactory system in golden Syrian hamsters. The olfactory epithelium (OE) is severely impaired and massive sustentacular cells were injured, causing numerous cilia loss (Bryche et al., 2020). SARS-CoV-2 may initiate its spread from the nasal mucosa and alveolar epithelium to the circulatory system and attacked cells in other tissues or organs by interacting with ACE2 or TMPRSS2 (Shang et al., 2020a).

On the other hand, SARS-CoV-2 can activate excessive and damaging immune inflammatory response in COVID-19, which is called “cytokine storm.” Mononuclear phagocytes are over proliferated with accumulated interleukin (IL)-6, IL-7, tumor necrosis factor (TNF), inflammatory chemokines CCL2, CCL3, and CXCL10 (Pedersen and Ho, 2020). Interferon regulatory factor 7 (IFN-7) may induce astrocytes to secret chemokines (Haddadi and Asadian, 2020). Extensive T cell responses during the cytokine storm could cause lymphopenia in patients. It is hypothesized that the cytokine storm may be induced by the inflamed nucleus tractus solitarius (NTS) in SARS-CoV-2 infection. Both hypothalamic/pituitary/adrenal axis and cholinergic anti-inflammatory pathway do not function in COVID-19 (Ur and Verma, 2020). The abnormal immune response following SARS-CoV-2 infection, in turn, induces autoimmune-related neurological complications. Severe lymphopenia and cytokine storm usually imply a poor prognosis of higher mortality (Tan et al., 2020).

Furthermore, the upregulated inflammatory factors and cytokines in serum can induce systemic hypercoagulable state. Thus, patients with COVID-19 are prone to thrombosis in both arteries and veins (Huang et al., 2020). In addition, increasing lipoprotein(a) (LP(a)) may promote the development of thrombosis in COVID-19. LP(a) could activate phospholipid oxidation to exert proinflammatory roles and split off the preformed atherosclerotic plaques. Besides, the endogenous fibrinolytic system is restrained by the apolipoprotein(a) compartments within Lp(a) molecules (Moriarty et al., 2020). The complicated metabolic mechanism still needs more exploration.

## Treatment and Clinical Management

To date, no specific drugs or vaccines for COVID-19 have been approved. Symptomatic treatment is performed on the infected organs and systems. For the severely or critically ill, mechanical ventilation and intensive care unit (ICU) admission are performed for life support (Alberti et al., 2020; Dalakas, 2020). Anticoagulant therapy is applied for patients with coagulopathy. Antimalarials are assumed to inhibit the release of pro-inflammatory factors in the CNS by crossing the blood–brain barrier with their high lipophilic properties (Ong et al., 2020). Serotonin, a classic neurotransmitter, could suppress the inflammatory response in CNS and PNS. Luis et al. reported that selective serotonin reuptake inhibitors (SSRI) might act as neuroprotectors and reduce neuropsychiatric disorders (Costa et al., 2020). A recent study found that fluoxetine could contribute to SARS-CoV-2 inhibition (Zimniak et al., 2020). However, inappropriate utilization of SSRIs may overactivate serotoninergic neurons and induce serotonin syndrome with lethal consequences.

Immunotherapies are conducted in the short term to moderate inflammatory responses after SARS-CoV-2 infection (Sharun et al., 2020b). Convalescent serum is used as a rapid therapy to induce passive immunity against COVID-19. Additionally, intravenously administered monoclonal immunoglobulins may block the activation of Fc receptors and reduce the cytokine storm in CNS during inflammation (Iqbal Yatoo et al., 2020). It could be an effective and safe treatment for COVID-19-related encephalopathy (Muccioli et al., 2020).

Corticosteroids and plasma exchange are applied to control the systemic overactivated immune response in severely ill cases with neurological complications (Sharun et al., 2020a). However, there are risks of exacerbated infection induced by immunosuppression. Recently, some monoclonal antibodies (mNA) have been postulated to neutralize SARS-CoV-2 via binding to its spike protein (Sharun et al., 2020b). Studies are required to explore the underlying mechanism in detail and propose a specific treatment with low side effects for COVID-19.

Scientists and researchers are working on various types of vaccines, including live attenuated vaccines; vaccines modified at the protein, DNA, or mRNA levels; and vaccines with or without vectors (Iqbal Yatoo et al., 2020). Great challenges still exist for the development of a stable and relatively long-term vaccine. Both effectiveness and clinical safety should be taken into consideration.

In order to control the source of infection and stop the transmission, strategies of isolation, quarantine, and physical distancing have been implemented. People are encouraged to use masks or face coverings and to follow hand hygiene practices (Rabaan et al., 2020). Other the other hand, the management and treatment of acute or chronic primary neurological diseases cannot be neglected (von Oertzen et al., 2020). Task forces comprised of medical staff have been placed to guarantee emergency care of acute neurovascular events (Al-Jehani et al., 2020). Clinicians can observe their patients with preexisting seizure disorders through regular telemedicine (Parihar et al., 2020). Moreover, because COVID-19 could cause neurological complications in children as well, pediatric neurology should pay more attention to their patients' manifestations for a timely diagnosis and treatment (Bonkowsky et al., 2020).

## Conclusion and Future Prospects

Since the outbreak of the COVID-19 pandemic, SARS-CoV-2 poses serious threat to both the CNS and the PNS (Wang et al., 2020). Although it is regarded to mainly affect the respiratory system, more and more neurological complications seem to be appearing which further deteriorate the patients' condition and prognosis. Those in severe or critical states from COVID-19 usually present with consciousness disorders caused by concurrent neurological deficits and require assisted ventilation. Despite intensive care, they may ultimately lose their lives. Aggressive supportive care, such as mechanical ventilation, reduction of intracranial pressure, or anticoagulation therapy, is necessary to improve the patients' systemic condition. On the other hand, mild cases may only present with olfactory or gustatory disorders, which can lead to missed diagnoses (Wang et al., 2020). Neurologists should explore the pathogenesis by CT or MRI scan and laboratory analyses, especially for CSF, since SARS-CoV-2 can affect the nervous system in various manners. The use of high-dose steroid therapy is always a challenge for patients with severe respiratory infections. Experimental treatments should only be undertaken to relieve a worsening critical physical status in patients with negative CSF results. Vaccines are imperative in this fight against the COVID-19 pandemic, but they still need massive research and developments.

**Figure 3:**
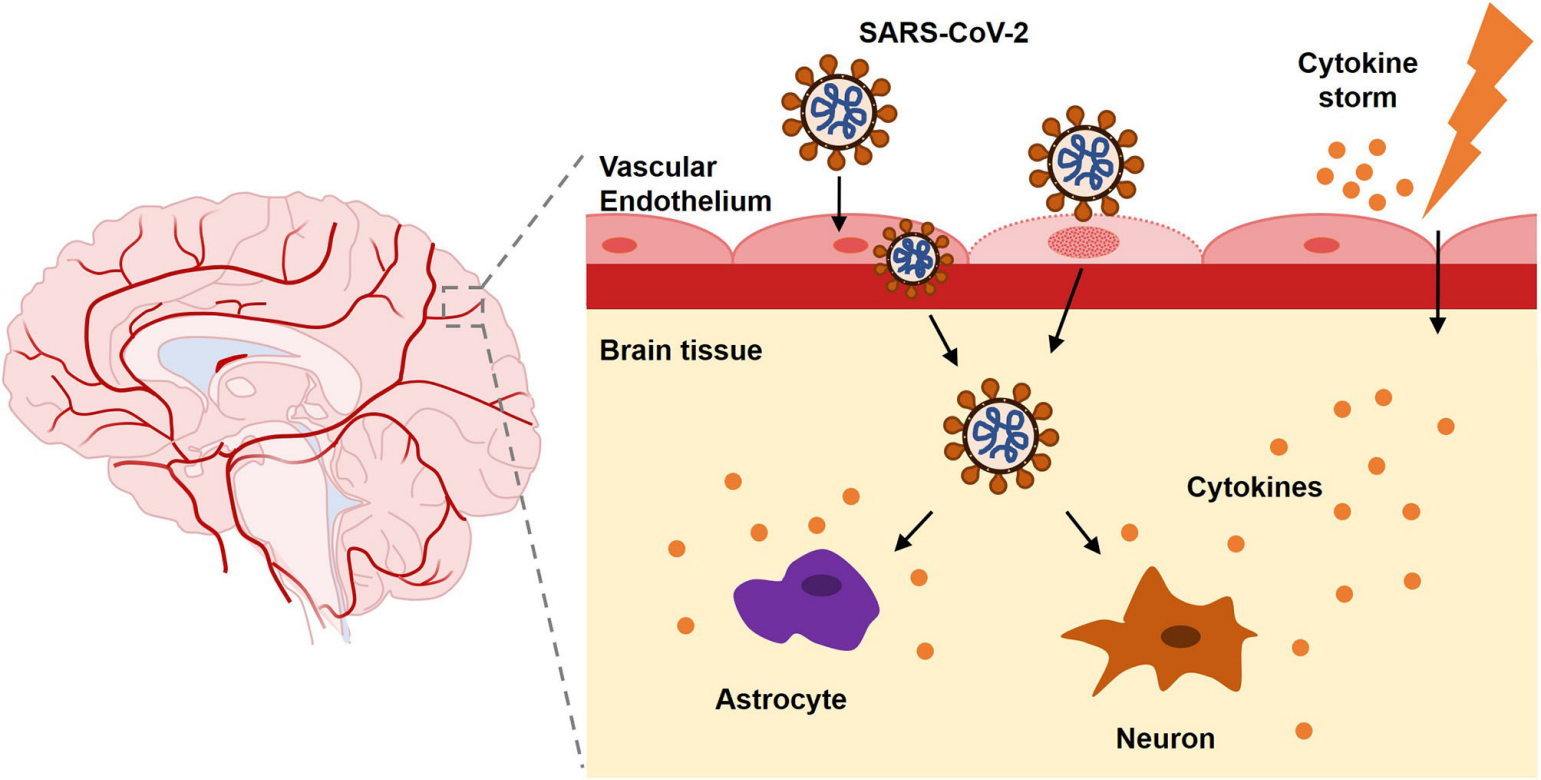
SARS-CoV-2 leads to neurological disorders via different pathways.

**Figure 4:**
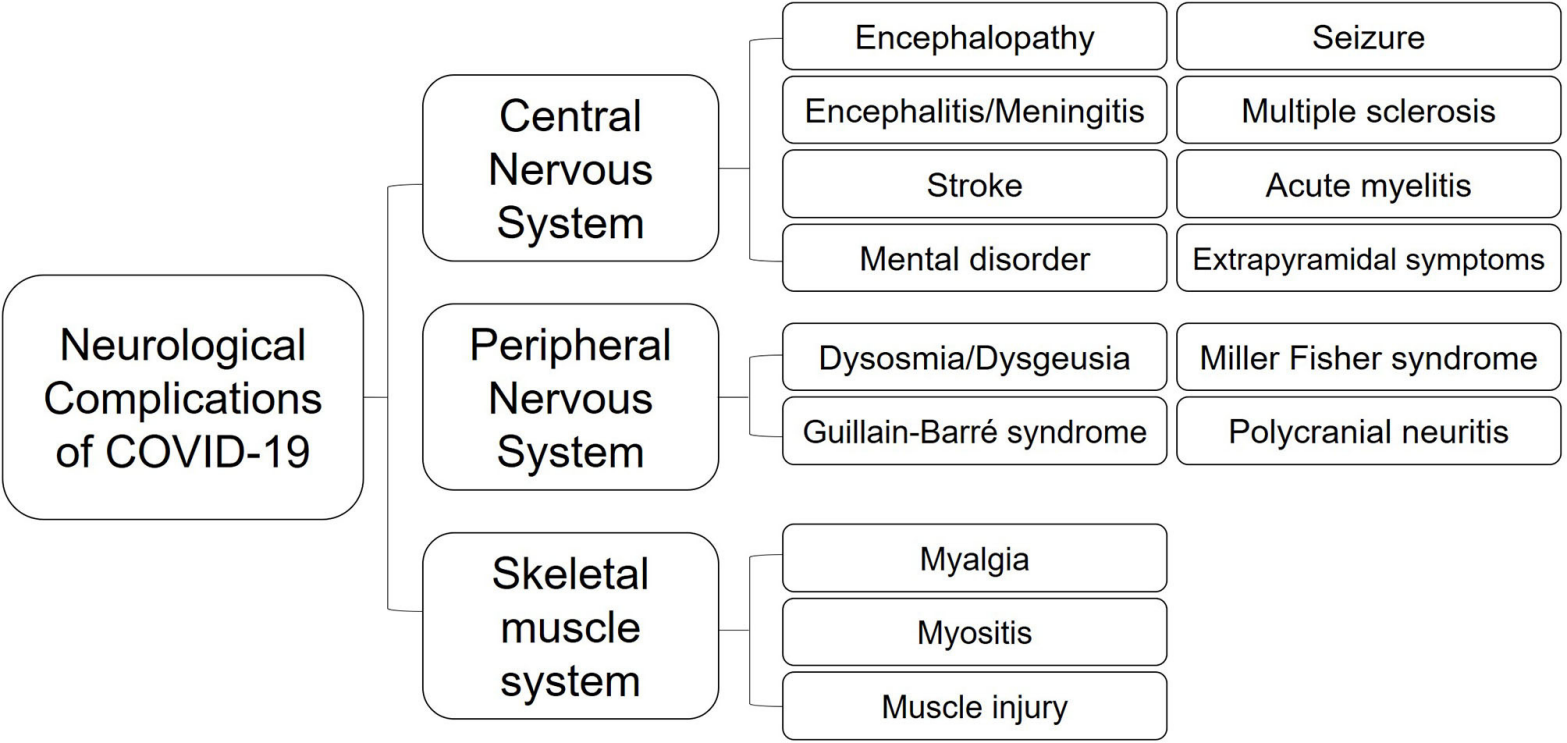
Neurological complications of COVID-19.

Since the virus can lead to multiple neurological disorders by different means (Figure 3), more studies are needed to determine the possible influences and moderating factors of SARS-CoV-2 infection. Early manifestations of neurological symptoms should be regarded as possible indicators of COVID-19 (Figure 4). Despite possible iatrogenic infections, individuals who feel unwell should seek medical attention immediately. Physicians who examine patients with atypical symptoms should ask for a detailed medical history and conduct the necessary PCR assays for SARS-CoV-2 to obtain an early diagnosis and avoid the wide spread. Further explorations are required on the molecular regulation of COVID-19 for a better understanding of SARS-CoV-2 neurotropism and its possible interferences.

## Author Contributions

SY designed the conception, searched the literature, draw the schematics, and created the manuscript. MY searched the literature, summarized the opinion, draw the schematics, and revised the manuscript. All authors contributed to the article and approved the submitted version.

## Conflict of Interest

The authors declare that the research was conducted in the absence of any commercial or financial relationships that could be construed as a potential conflict of interest.
